# Sensor Node Network for Remote Moisture Measurement in Timber Based on Bluetooth Low Energy and Web-Based Monitoring System

**DOI:** 10.3390/s21020491

**Published:** 2021-01-12

**Authors:** Mohamed Saban, Leandro Daniel Medus, Silvia Casans, Otman Aghzout, Alfredo Rosado

**Affiliations:** 1 Department of Electronic Engineering, ETSE, University Valencia, Av. Universitat, s/n-46100 Burjassot, Valencia, Spain; leandro.d.medus@uv.es (L.D.M.); silvia.casans@uv.es (S.C.); alfredo.rosado@uv.es (A.R.); 2 Department of Computer Science Engineering, SIGL-Lab, ENSA, University Abdelmalek Essaadi, 93153 Tetouan, Morocco; oaghzout@uae.ac.ma

**Keywords:** moisture estimation, wood moisture sensor, IoT, BLE, cloud server, IoT network, web application, SoC

## Abstract

This paper proposes an IoT system based on wireless BLE connectivity to monitor the moisture content of wood, using a compact and low-cost moisture device that relies on a resistance measurement method valid for an ultra-wide range of resistance values. This device is digitally controlled with a BLE-incorporated micro-controller characterized by its small size and low power consumption, providing long-life battery. The proposed system consists of two main parts: first, the BLE moisture device including the moisture content measurement and wireless capability (BLE); second, the cloud-based monitoring platform, providing remote visualization and control for all the sensor nodes of the network. The complete infrastructure shows how multiple nodes can read and transmit moisture content of timber in buildings using small and unattended devices, with data saved in a central database and monitored by multiple commercial devices such as PC, smartphone, tablet, etc. The proposed system is innovative, scalable and low cost, and it can be deployed in wooden buildings and the wood industry, providing a practical solution that will help to avoid rot and other damaging effects caused by the moisture content.

## 1. Introduction

The internet of things (IoT) allows the connection of devices to establish a strong infrastructure to control and monitor real-world physical systems [1,2]. An IoT system consists of many devices connected to a gateway or multiple gateways, as an intermediate between the devices and the server equipped with an IoT platform [3]. IoT offers smart devices to automate homes, and it is also essential to business: IoT affords a real-time control of their systems and allows companies to automate processes and reduce labor costs so it can help saving money and time, generating more revenue. In the literature, many works implement the IoT as a solution responding to several problems in the different fields such as smart agriculture [4], healthcare services [5], fire detection [6], water monitoring [7], etc. Also, due to the actual situation of pandemic, the authors in [8] provided an IoT system to automate health monitoring. Sensor nodes can be used for continuous sensing, “e.g., location sensing”, the micro-sensing concept and the wireless connection of nodes promise many new application areas in all aspects of life. In this work, we present a secure IoT-enabled smart system that allows the monitoring of the moisture of wood. It is well known that the moisture content is one of the most important factors altering the properties of wood: the weight and the strength of wood can be affected due to the contained amount of water, thus it becomes weak and more susceptible to biological attacks [9]. Presently, the measurements of the moisture content are done manually with huge devices that always require a human interaction. Christian in [10], proposes a system for long-term recording of wood moisture content with internal conductively glued electrodes. Moron in [11] presents an application of the measurement of wood moisture in the restoration of old buildings.

Our proposed system can be used in different scenarios:

- Smart Home: The system can be used for wooden houses or structures to anticipate damages in case of water leak, fungi, or any alteration leading to wood deterioration.
- Cultural Heritage: The system can be used by maintenance services in old buildings made of wood (churches, museums, palaces) since the moisture content is the most important factor that can affect the wood strength.
- Industry: Measuring the moisture content of wood correctly is an important key in the wood processing industry, “e.g., veneer manufacturing: the gluing quality is affected by moisture when multiple veneer sheets are glued to form plywood”.

As we know, Bluetooth Low Energy (BLE) is a wireless technology developed by the Bluetooth Special Interest Group (SIG) for short-range communication [12]. In the literature we can find some papers where BLE was used as a communication protocol. Ensworth in [13] shows how back-scatter signals in an IoT system can be designed for compatibility with the BLE chipsets. Also, Luo describes in [14] a scheme for BLE-based IoT network.

The authors have previously developed [15] a device with a novel and accurate resistance measurement method: the resistance value of any material or substance can be obtained and then converted into a physicochemical magnitude “e.g., moisture content in percentage”. In this paper, this measurement device was adapted to build an IoT network system for remote monitoring of the moisture content of wood through a web application running on computers and smartphones.

Most of the time, when sending small amount of data in a short communication range, it is better to use Zigbee or Bluetooth instead of Wi-Fi because of their low power consumption, [16]. Comparing the energy consumption of BLE and Zigbee, BLE consumes less [17] and consequently, it offers very low power consumption which lead to a long node battery life [18]. In addition, Bluetooth is incorporated in daily used devices such as laptops and smartphones, not requiring any additional adaptor or hardware to connect to the IoT system. In this work we decided to adapt the previous device to BLE technology as an alternative to Wi-Fi.

The complete system interface for management and monitoring is fully developed as a web application using PHP programming language, one of the most popular and efficient dynamic language for web applications [19]. Users can access the data and use it without limitations anywhere and anytime. The web application is running in Apache web server environment with MySQL database management for the server. Database MySQL gives better performance for small data and simple queries [20]. The database can store unlimited data depending on the storage available in the server because the effective maximum size of a table is determined by the operating system constraints on file size and not MySQL limits [21]. In addition to its speed and reliability, MySQL server provides strong data protection [22].

The main novelties of this paper can be summarized in the following points:

- Development of a smart and accurate BLE moisture sensor controlled remotely.
- Development of a secure web application with authentication system. This application allows the users to build their networks, and control the BLE sensor nodes.
- Deployment of a scalable and low-cost system for indoor uses with the aim of monitoring the moisture content of different types of wood. This system consists of a central server, multiple gateways and BLE sensor nodes. The whole system is managed remotely through the developed web application, using commercial devices such as smart phones and laptops, etc.

The objectives of this work are detailed in Section 2, Section 3 describes the BLE protocol and how BLE devices communicate with one another. The circuit of the developed BLE sensor node is represented in Section 4 describing its components and characteristics, in Section 5 a test bench was built to get experimental results of the BLE sensor node. Section 6 describes the web application development, its contents and functionalities, and the connection between the cloud server, the gateway and the web application. Lastly, conclusion and future work are presented in Section 7.

## 2. Objectives

The main objective is to build a wireless sensor network, consisting of spatially distributed autonomous moisture devices to monitor wood conditions. A gateway collects data from the connected BLE sensor nodes, process the received information and transmit the essential information to a cloud server using Ethernet (wired or wireless). The gateway also receives data to control nodes.

The following goals were proposed:

- Sensor nodes: Create new BLE capable sensor node by designing an electronic circuit in the measurement device to replace Wi-Fi protocol, including a new printed circuit board (PCB).
- Gateway: Build a complete BLE wireless sensor network using the newly created sensor nodes. Each sensor node will transmit the measured values (resistance and moisture) Remotely to a gateway that will store these values in the remote server database. Each gateway may correspond to a different monitoring area: a building, a house, a timber stock, etc.
- Remote server: Create the database infrastructure for data receiving and data transfer between users and devices.
- User interface: Develop a web-based application, with the aim of monitoring and controlling the sensor nodes and analyzing their data. The application must allow users to manage gateways and sensor nodes, check the measured values in each node, in the form of charts and tables that can be downloaded from the web application. Additionally, an administration level must be included to manage users.

**Figure 1:**
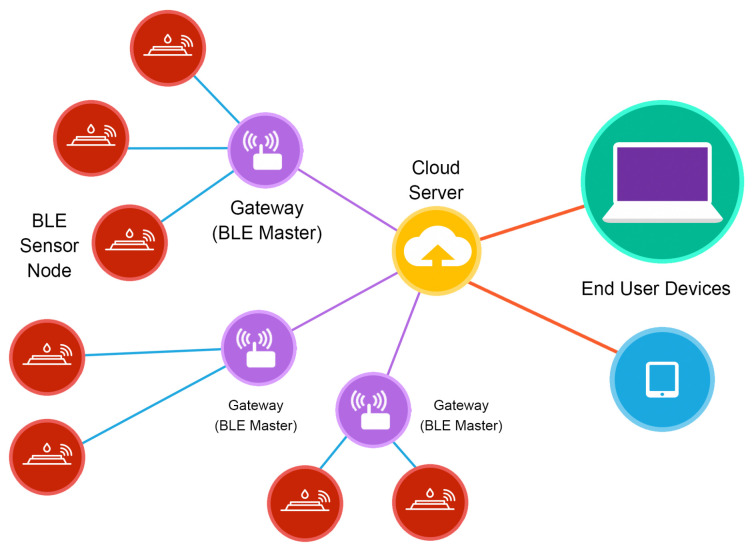
The proposed BLE-based wireless network topology: BLE communication between sensor nodes and gateways. Gateways are connected to the cloud server via Ethernet. Users can control/monitor nodes using the web application.

The proposed network architecture, described in Figure 1, is composed of several BLE sensor nodes connected to a central gateway, each gateway is connected to the cloud server database via Ethernet or via Wi-Fi. This centralized architecture allows use of the same cloud server for different group of devices. The sensor nodes can continuously monitor environments with low power consumption. The BLE master node (the gateway) receives and processes data before sending it to the cloud and allows sending user or system commands to the nodes through the web application (user interface).

## 3. Ble Protocol

**Figure 2:**
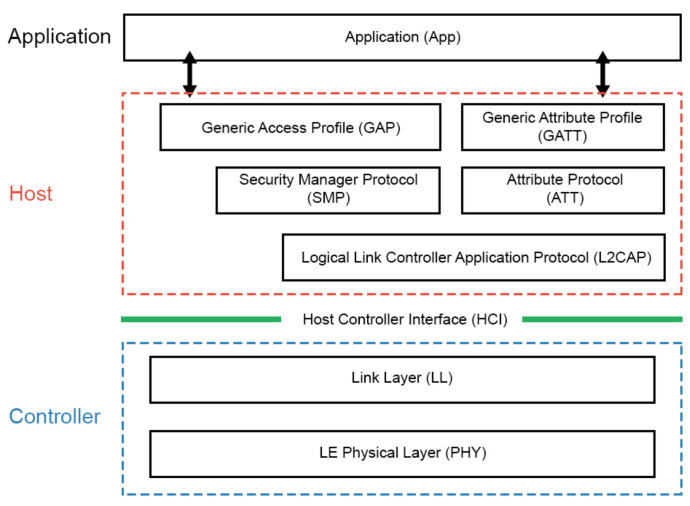
BLE protocol stack. The BLE stack consists of two parts: Host and Controller [25].

BLE is a low energy version of Bluetooth specified in version 4.0 [23]. Presently, it is widely used and comes incorporated in most smartphones and other smart devices [24]. Figure 2 represents the BLE protocol stack, this protocol is partitioned into two independent main parts: the ’Controller’ and the ’Host’. The controller part runs the Physical Layer that transmits and receives bits, and the Link Layer handles physical layer packets and all associated timing. Generally, it is implemented in the form of a small SoC (System-on-Chip) with an integrated Bluetooth radio. The link layer provides connection establishment, flow control and error control.

The host contains the upper layers:

- L2CAP (logical link control and adaptation protocol): defines the generic procedures associated with the discovery of BLE devices, it provides fragmentation and reassembly for vast data packets and multiplex the data channels from the upper layers.
- GATT (generic attribute protocol): GATT defines how the devices communicate between each other, a BLE device can be GATT server or GATT Client.
- GAP (Generic access profile): defines how BLE devices discover each other, establish a connection and interact based on their roles (Observer, Peripheral, Broadcaster or Central).

**Figure 3:**
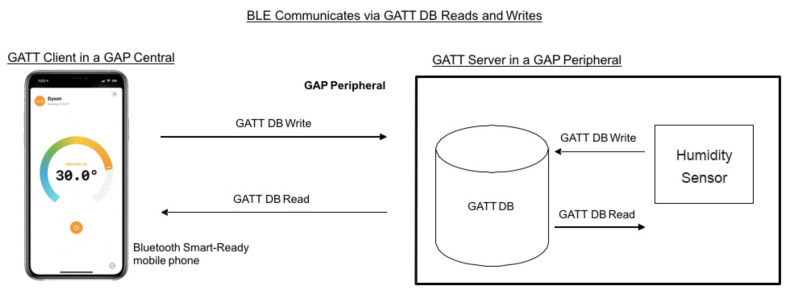
BLE Communicates via GATT DB, where GATT server is the humidity sensor and GATT client is the mobile phone.

Figure 3 shows an example of a server that runs a ‘Humidity sensor’ service. The GATT database (GATT DB) stores and provides data, it runs in a GAP Peripheral and responds to read and write requests from both GAP Central and the GAP Peripheral itself.

Like classic Bluetooth, BLE uses adaptive frequency hopping spread spectrum to access the shared channel [17]. BLE provides, approximately, a 3 dB better link compared to Classic Bluetooth [26]. A BLE device can operate either in master or slave role. A master can manage many simultaneous connections of slave devices, but a slave can be connected only to a single master. BLE networks are built with a star topology. Differently from classic Bluetooth, the BLE slave advertises on one or several of the three channels designated for advertisement while discovering.

Encryption in Bluetooth low energy uses the Advanced Encryption Standard (AES) operating with Cipher block Chaining Message authentication code (CCM) mode. AES-CCM cryptography has 4 inputs: AES key, a nonce, plain-text and AAD (optional additional authenticated data). This cryptography generates 2 outputs: a cipher-text and a message authentication code (an authentication tag) [27]. The BLE Controller perform the encryption function by generating 128-bit encrypted data from a 128-bit key, and 128-bit plain-text data using the AES-128-bit block cipher as defined in the Federal Information Processing Standards Publication FIPS-1971 [28].

## 4. Circuit Development

The moisture sensor device described in [15] was formed of two main parts: analog part containing the amplification and filtering section, responsible for resistance measurement; and a digital part that reads the sensed values from the analog part and transmits them via Wi-Fi. The analog part and the digital part were designed as two separated boards to minimize interference, and to make the design more flexible.

In this work we designed a new PCB for the digital board to support BLE using the same analog board of the old device. The measurement algorithm for the new digital board takes the average of 50 instead of 10 in the old one. In addition, new functions were added for the BLE communication along with a new function for error detection. This function allows the device to measure again if the obtained value is not correct. The algorithm uses a conversion formula based on the physicochemical properties of the wood to convert the measured resistance into a moisture value. The types of wood are selected by the user in the web application. This device provides accurate measurements with a relative error lower than 1% as described in Table 1.

The principle component of the new board is the CYBLE-012012-1 SoC from Cypress. This module contains a 32-bit processor (0.9 DMIPS/MHz), operating at up to 48 MHz. It was selected because of its small size of 14.52 mm × 19.20 mm × 2.00 mm, and the possibility to connect up to 23 GPIOs (general purpose input/outputs) configurable as open drain high/low, pull-up/pull-down, which allows the connection of other electronic boards. It includes Bluetooth 4.1 qualified single-mode module. The power consumption and transmission characteristics of the module are shown in Table 2.

**Figure 4:**
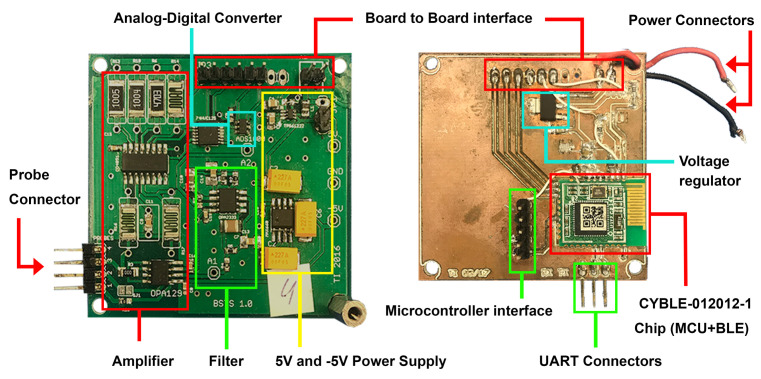
BLE moisture device circuit: analog and digital boards. The size of the circuit when the boards are connected: 5.7 cm × 5.3 cm × 1.5 cm.

Figure 4 shows the BLE device circuit. The interface between the two boards, analog and digital, is consisting of 8 pins: 2 ground pins, I2C bus interface for A/D converter, 3 analog multiplexer control lines to control the gain, and one analog power shutdown line to minimize power consumption by switching on/off analog power supply. All lines of this interface are 3 V. The new digital board replicates the Wi-Fi board communication lines with the analog board but providing a new remote communication method. Thus, no redesign of the analog board is required.

The BLE SoC is powered through the low-drop voltage regulator AMS1117, providing a stable voltage of 3 V to its components. This SoC is a complete RF solution and communication stack for Bluetooth Low Energy data sharing. The module also supports several peripheral functions such as ADC, timers, counters, I2C communication, UART and SPI through its programmable architecture. The device was developed to read the resistance values from the analog part, processing the information gathered and notifying another device over BLE. The instant resistance value is obtained by calculating the average of the last 50 measurements (averaged over a sliding window), and by using pre-calculated property-resistance, the device obtains the physicochemical value of the material. The type of the material (and consequently, the corresponding conversion and correction curves) can be selected by the user remotely through the web application.

**Figure 5:**
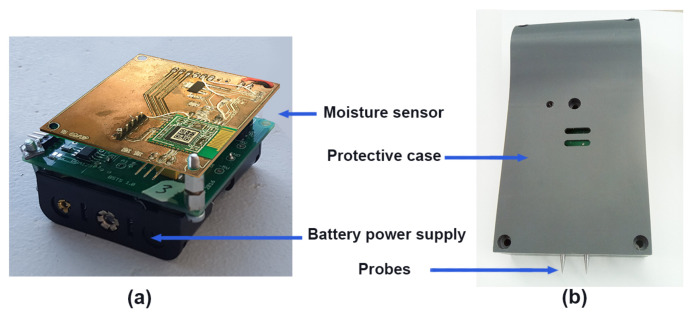
The BLE sensor node prototype; (**a**) the BLE sensor circuit with battery power supply, (**b**) the protective case of the BLE sensor with the measurements probe.

To make it portable, additional circuitry for battery power supply was integrated with the BLE sensor as described in Figure 5a. The BLE sensor is alimented by three AAA 4.5V batteries. The size of the whole circuit is 5.7 cm × 5.3 cm × 3 cm. A protective box was designed to cover it, the probes are stainless and the protective box is made of epoxy material. Thus, no corrosion problems deriving from the continuous use are expected. The prototype of the final product is described in Figure 5b.

**Figure 6:**
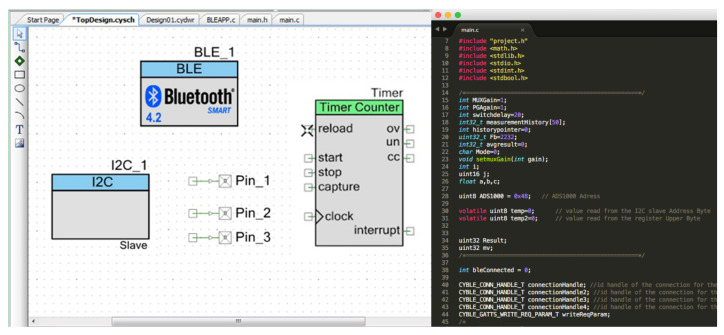
Screenshot from PSoC Creator IDE software showing the final BLE application schematic and a part of the main algorithm.

The accompanying software ’PSoC Creator IDE’, makes it easy to develop and program a BLE application. A block diagram of the components activated from the PSoC Creator software is displayed in Figure 6: An I2C block reads ADC, BLE block manages BLE communication, 3 pins for the multiplexer and the timer block controls data sample rate and wake-up/standby. Adding those components allows use of their functions in the main code.

**Figure 7:**
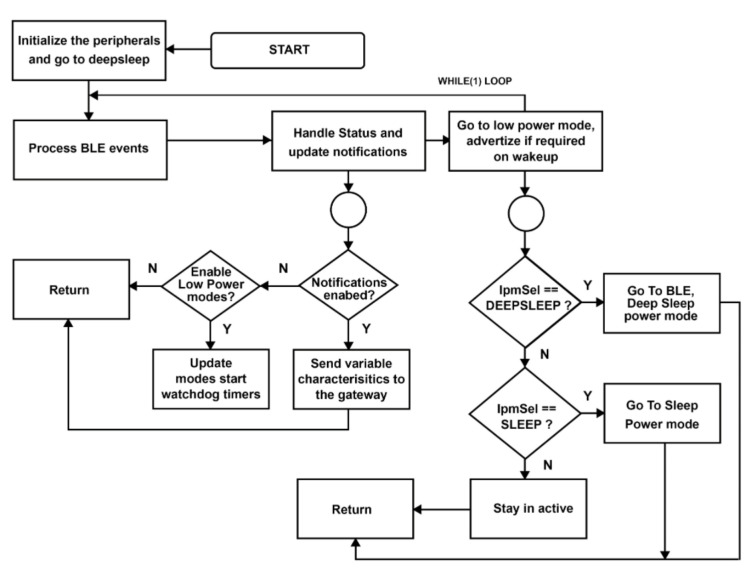
The simplified diagram flow of the deep sleeping mode process configuration of the BLE sensor node.

The developed BLE moisture sensor is battery operated. A long battery life is a key requirement for such devices. The device is programmed with a low-power solution as shown in the diagram flow represented in Figure 7: the device is programmed to send the data to the gateway and enter deep sleeping mode and wake up in the next notification from the gateway. This deep sleeping mode allows the BLE device to minimize the consumption of energy and keep the battery life for a long time.

As summary, the sensing device is characterized by its small size and accuracy with the following specifications:

- Ultra-wide measurement range: can measure resistances values from 1 MΩ to 100 GΩ.
- Communications: wireless data transmission via BLE and possibility to attach a local display for visualization options and control.
- Portability: Low power consumption, lightweight and small size.
- Flexibility: The circuit can be customized for different application areas by adapting firmware/software. Consequently, the same electronic system can be used for multiple purposes related to resistance measurement.

## 5. Experimental Results for the Ble Sensor Node

**Figure 8:**
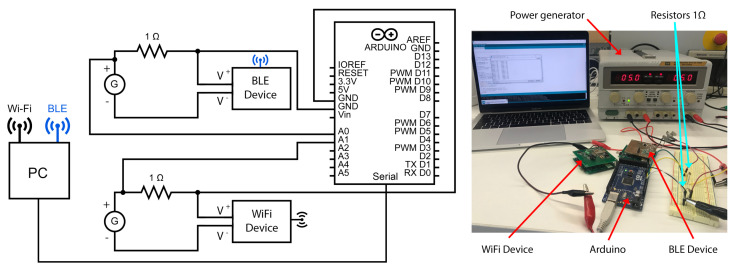
Test Bench: The BLE device is connected to the computer via BLE, and the Wi-Fi device is connected via Wi-Fi. Both devices are connected to a power generator with fixed voltage, a 1 Ω resistor and an Arduino micro-controller allows measurement and recording of the flowing current into both devices. The PC reads the power consumption from Arduino.

A comparison between the Wi-Fi sensing device and the new BLE device is done to verify the functionality and compare the power consumption of the two devices. The chip used in the Wi-Fi device is ESP8266-Wi-Fi Module, and the chip used in the BLE device is CYBLE-012012-1. A measurement circuit was installed, consisting of a resistance R = 1Ω ± 1%, and an Arduino micro-controller programmed to record the electric current flowing into the devices. Both devices are powered with a stable voltage $V_{i}$ = 5 V (Figure 8). Each device is connected to the PC using their respective wireless communication method. A Python script is implemented in the computer to read the values measured with the two devices, both devices are programmed to enter sleeping mode when there is no data transmission. Figure 8 shows the test bench with the schematic.

**Figure 9:**
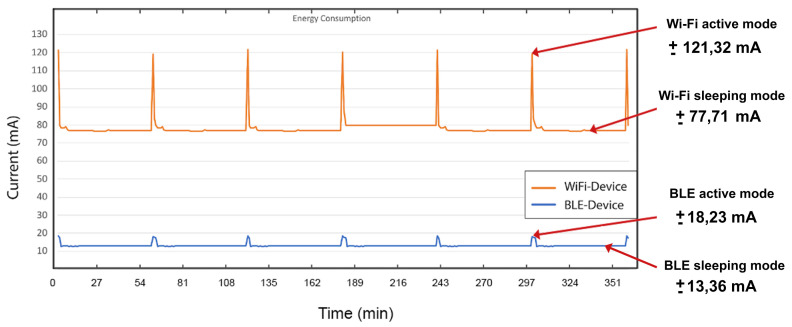
The current flowing into the devices as a function of time. The Wi-Fi board shows higher consumption in active and sleeping mode.

Figure 9 shows the current flowing into the BLE device and Wi-Fi device for 6 h. From the recorded data during the operation of both devices, the power consumption can be obtained by calculating the average of the flowing current. In this specific scenario, the devices are programmed to send new values every hour. The BLE device approximately consumes 13.36 mA, while the Wi-Fi device consumes approximately 77.71 mA. As summary, the proposed BLE circuit can send the data to the gateway using 82% less power than the Wi-Fi circuit.

A second test was done inside the electronic department of the engineering school ETSE with the aim of measuring the maximum transmission distance allowing the BLE sensor to send and receive data correctly from the gateway in an indoor environment.

**Figure 10:**
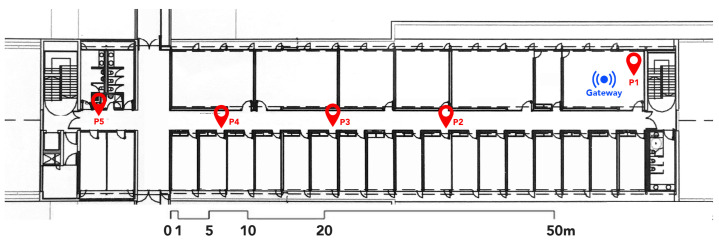
Plan of the Electronic department of ETSE (Univ. Valencia): the gateway is placed inside our laboratory. P1 to P5 are the different positions of the BLE sensor node.

Figure 10 shows the plan of the department where the gateway is inside our laboratory, and the BLE sensor was placed in different positions (P1 to P5). Table 3 describes the distance between the measurement positions of the BLE node and the gateway, the received signal strength indication (RSSI) and the signal quality. The ’CYSMART BLE Test and Debug Utility’ software from Cypress was used to transmit data and get the results described in this table.

This test shows that the BLE sensor node can achieve a transmission distance of 71 m without any additional power amplifier. Low signal quality causes lack of data reception, the position P5 was the last position where we could send and receive the data correctly. Any signal that exceed −92 dBm is unusable. Walls and different obstacles cause blocking effects between the gateway and the sensor node, for that it is useful to evaluate the blocking effects before deploying this system to get an appropriate placement of the sensor nodes, and consequently getting a better signal coverage to obtain a reliable and robust IoT network.

## 6. Web-Based Monitoring System

Research of web monitoring system becomes popular with the widespread use of IoT sensors. Awaj [29] proposed a temperature and humidity acquisition system in server room, this web application was developed using simple HTML with acquisition data viewer without authentication system. Wibowo [30] proposed a development of embedded gateway for wireless sensor network and internet protocol interoperability, his application was developed using PHP and Javascript, to script a responsive page, it uses Bootstrap framework implementing AJAX. Alfat [31] described an implementation of Laravel framework in web-based temperature and humidity Monitoring System. A similar approach is used in this work using different functionalities and structure.

### 6.1. Architecture of the Monitoring System

**Figure 11:**
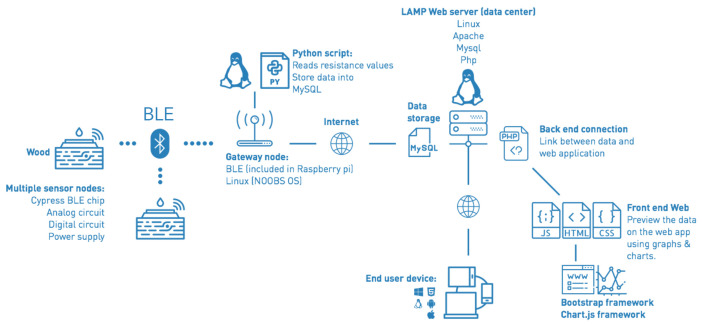
Project diagram, sensors send the measured values of moisture via BLE to the gateway, the remote server receives the data from the gateway via Internet and store it into its database. The user interface is connected to the database to monitor and control the sensor nodes.

The proposed monitoring system is represented in Figure 11, it is flexible so it can be applied for different functionalities, it can be updated based on the operator’s need. The gateway uses Python scripting for BLE communication, data analysis and Ethernet data exchange with the server. A remote server was configured with LAMP (Linux, Apache, MySQL and PHP) software and prepared to host the web application.

We can divide this diagram into two section:

- the first section consists of the BLE sensor nodes and the gateway. A Raspberry Pi 3 is used as a gateway to send system command to the sensor nodes, and receive data from the sensor nodes convert it into float values and write them into the server’s database via HTTP protocol. A Python script is used in the gateway to wake up each BLE device to send new values respecting the period of time defined by the user. This period is defined while configuring a device in the setting page of the web application. This script processes the data for error detection before sending it to the cloud server. Also, it checks the connection between the gateway and the sensor nodes of the network. In case of losing the connection, the gateway sends an error message to the server, and the user will be notified. The developed system is dedicated to indoor use, it will handle a small number of devices due to the range of the communication. The actual gateways have a RAM memory of 1GB LPDDR2 (900 MHz), which is sufficient to handle the connected sensor nodes in the local network taking into consideration that the sensor nodes are not sending data all the time. Thus, we do not expect any memory limitations.
- The second section is consisting of the cloud server and the web application. The cloud server is using Apache web server environment with MySQL database management. This environment is based on a multi-process (MP) architecture which spawns many processes to support many clients [32]. The web application was developed using PHP programming language, one of the most popular scripting languages used in Web development [19]. Users have access to the data without limitations, they can used it anywhere and anytime.

**Figure 12:**
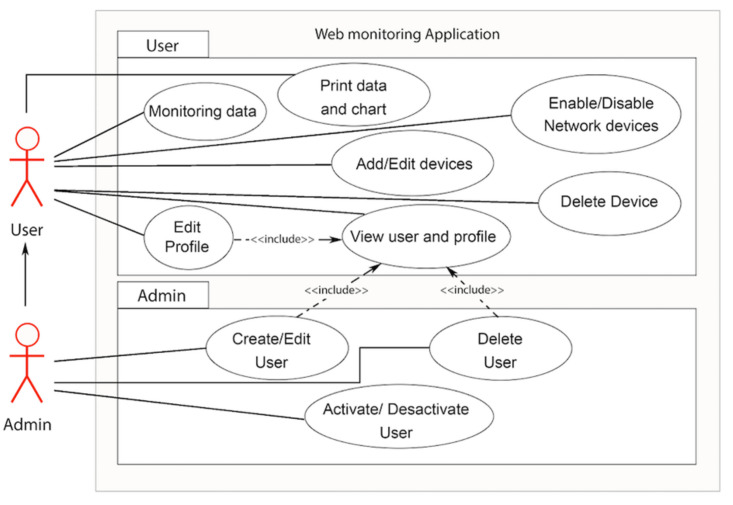
Web application use case diagram.

In this paper, the web application will be represented through the object-oriented modeling diagram. Modeling is a central part of all the activities that lead up to the deployment of good software. Unified Modeling Language (UML) was selected as modeling tool to describe the structure of the system in Figure 12.

The user has a parent role of administrator. The user role can create a new network and add/update/remove gateways and devices to this. The user can monitor data also export it into an Excel or CSV file which can make data analysis easier. The administrator role can add, update and delete users and manage their networks.

**Figure 13:**
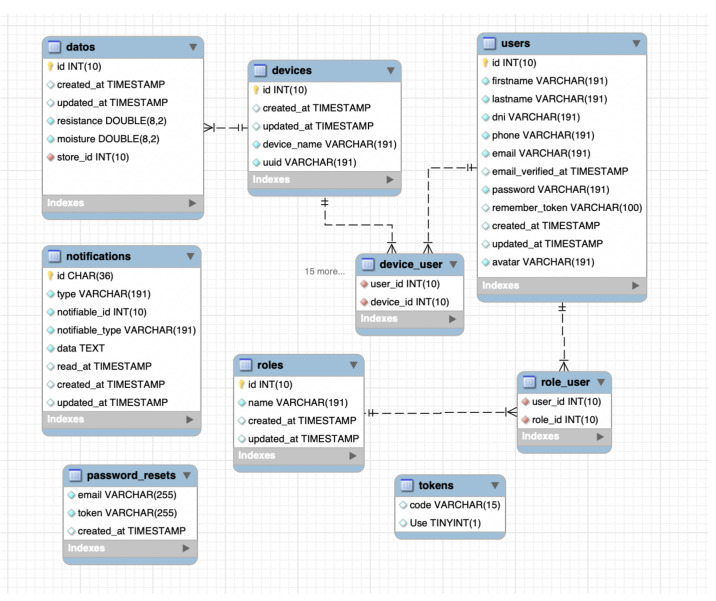
Web application Database design represented by EER diagram.

The database design is represented in Figure 13 through its EER (Enhanced entity-relationship) diagram. The database is consisting of 6 tables: ’Users’, ’Devices’, ’Notifications’, ’Roles’, ’Datos’, ’Password_reset’, and 2 pivot tables: ’Role_user’ and ’Device_user’. The ’Users’ table roles: administrator or user. Each user has associated gateways and devices, where the data received from each device is stored into ’Datos’ table. The ’Notifications’ table will store the notifications generated by the application when a device receives a value out of the range specified by the user in setting Section 6.1.

The Twitter Bootstrap framework was used to develop the Front-End of the web application, this framework provides a set of JavaScript functions and CSS classes making the process of front-end development easier. This framework provides a responsive design which enable support for many mobile devices, and tablet and desktop interfaces [33].

**Figure 14:**
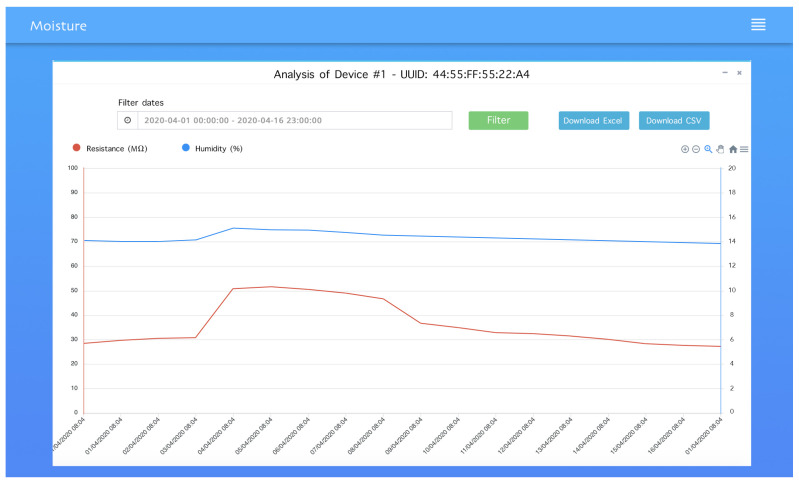
Web application device dashboard page showing the chart of moisture and resistance in function of time, the user can filter data by date and download it as Excel or CSV file.

The dashboard user interface is described through one screenshot in Figure 14. The web application has six menus: ’Home’, ’Setting’, ’Dashboard’, ’Profile’ and ’Notifications’. In the home page, the user can see his networks and its devices (gateways and sensor nodes). when the user clicks on a device he will be redirected to its dashboard page where he can check the data charts and export them. The setting page allows users to add new devices including gateways and sensor nodes, he can edit, delete and activate or deactivate a device. In this page, the user defines limits values for each device, if the data received is out of the specified range, a notification will be generated by the application and shown on the user profile, and it will be sent via email through SMTP protocol [34]. In the profile page the user can update his data, and notifications page allows the user to check all the notifications received.

### 6.2. Experimental Test Bench for the BLE Network

**Figure 15:**
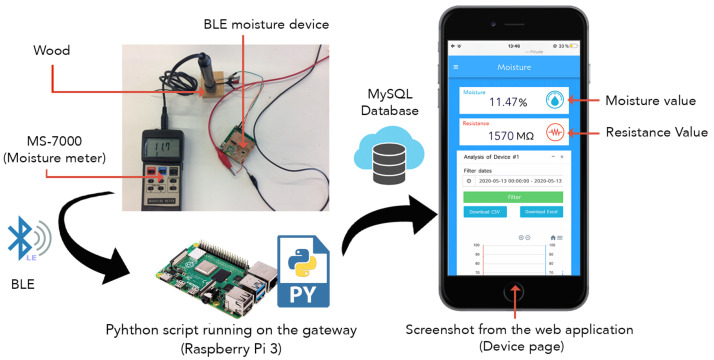
Testbench used to check the functionality of the system. The BLE device is connected to a Raspberry Pi 3 through BLE. The Raspberry receives the values, convert them to float and write them into the server database. The user can check the last stored values through the web application.

To verify the functionality of the system, a benchmark experiment was conducted with two main aims: flexibility, performance, speed of the web application and adaptation to the BLE device. The BLE device is powered at 5 V and connected to the gateway through Bluetooth Low Energy. The gateway writes the received values into the cloud server database after converting them. To compare the results, we used the moisture meter ’Lutron MS-7000’ to measure the moisture content simultaneously. This digital meter has 9 material species groups in memory, making it suitable for measuring moisture. It uses 2 pins electrode to measure moisture content of the wood (show in Figure 15).

The previous test was repeated using various types of wood, the results obtained by the moisture meter MS-7000 are approximately the same as the results measured with the BLE device. The test was done at 25 ºC. Table 4 shows the obtained results.

From the previous results, we can see that the obtained values are very similar to those measured by the moisture meter MS-7000. The deviation is acceptable, because the calculation method of the developed sensor and the moisture meter are different. Also, the moisture meter support only 9 registered wood materials, while our device is supporting 36 different materials. For the moisture meter we had to select the type of material for each piece of wood. In contrast, the BLE device can supports more materials which can be added by the user through the setting page on the web application.

The time between sending a value from the BLE device and storing this value into the server (MySQL database) is between 2.1 and 3.4 s. The web application is configured to read values from the database and refresh the displayed results every minute, this timer can be changed depending on the use of the application, for example if we want real-time values we can set it to 1 s, but in the case of measuring moisture content we do not need to monitor the values in strict real time, thus, we reduce the use of server resources. The web application has been tested with multiple resolutions, various web browsers, tablets and mobile phones showing good responsiveness.

The authentication system provides the privacy and privilege control for users. Users can configure the periods of receiving data from each device through the web interface. in case of losing BLE connection between a device and his gateway, the web application will show a warning message in the dashboard and send an email to the user (the owner of that device) to notify him that a problem exists. In case the gateway loses the connection, a warning message will appear in the user dashboard to notify that his gateway is not connected to the Internet. The gateway will use an internal SD card memory to store the received data from the BLE devices, and once the connection is back, the gateway will write the stored data into the server database.

In summary, the proposed device can accurately measure electrical resistance values that are converted into moisture values. This conversion procedure is programmed in the BLE SoC in the digital board; thus, the new digital processing techniques can be included by updating firmware.

## 7. Conclusions and Future Work

This work proposes Bluetooth Low Energy communication as an alternative to Wi-Fi for the wood moisture measurement device. Tests show that the new device consumes 80% less power than the old one, keeping the same performance and accuracy. To make it portable, the new circuit is completed with additional circuitry for battery power supply.

The proposed monitoring system can be used in old churches, where the roof and walls are made of wood, also, for wooden buildings to control the content of moisture to face problems of biological attacks. In the industry field, it can be used to measure the correct moisture content of wood during the fabrication process.

The strengths of proposed system:

- The cost: the fabrication of the BLE sensor circuit is not expensive. The price of the final product is very cheap in comparison with the manual one, also the gateway used is a raspberry pi which costs around 30€.
- Central topology: one central server is managing all the system’s transmitted data, this topology has lower hardware and support costs. It is faster to deploy and easier to manage because messaging architecture is simplified: no need to coordinate installation among geographically distant sites. This architecture supports the deployment of large number of BLE devices and gateways which allows a big number of users to use the same server and web application.
- Local data storage: the gateway stores the data into the internal memory before sending it to the cloud server, this process helps to not lose the measured values if there are some connection failures between the gateway and the server.
- Notifications: the users are notified if the value of the measured moisture is out of the range specified previously, also when the connection is lost between the devices and the gateway or between the gateway and the server.
- Authentication system: all the routes leading to the user data are protected. Only the authorized person can access his account to add/remove devices and monitor his data.
- UI/UX design: the user interface design (UI) of the web application is beautiful. It is easy to use and respect the user experience (UX). The design is responsive, and the application works perfectly across all the devices and web browsers.

As mentioned in Section 5, The connection between the cloud server, the gateway and the BLE devices is established using a Python script launched in the gateway. To execute this script, the user should open an SSH (Secure Shell) connection with the gateway and launch the script via the terminal. The next step of this work is to develop a user interface for the gateway to make the previous task easier for normal users. This interface will show the connected nodes, the connection status with the cloud server and display errors. Always using a Raspberry Pi as a BLE gateway, the user can access this interface by attaching a screen to Raspberry Pi via HDMI. New features will be added to the web application to make it more stable, faster and robust.

## Figures and Tables

**Table 1:** Quantitative table: the nominal value of the measured resistance (Rn), the calibrated resistance value (Rc), the obtained value using the sensor (Rm) and the relative error (ξr) when comparing the results with the calibrated value Rc.

Rn	Rc	Accuracy (%)	Rm	ξr (%)
1 MΩ	1.0000057 MΩ	±0.00002	1.0049 MΩ	0.5
10 MΩ	10.000028 MΩ	±0.0005	10.083 MΩ	0.8
100 MΩ	100.00173 MΩ	±0.01	100.4211 MΩ	0.4
1 GΩ	1.000183 GΩ	±0.005	1.0042 GΩ	0.4
10 GΩ	10.016717 GΩ	±0.005	10.0436 GΩ	0.3
100 GΩ	99.89014 GΩ	±1	99.235 GΩ	0.7

**Table 2:** Power consumption characteristics of the module CYBLE-012012-1.

TX output power	–18 dbm to +3 dbm
RX output power	–18 dbm to +3 dbm
TX current consumption	15.6 mA (radio only, 0 dbm)
RX current consumption	16.4 mA (radio only)
Low-power mode support (Deep Sleep)	1.3 µA with watch crystal oscillator (WCO) on
Low-power mode support (Stop)	60 nA with GPIO (P2.2) or XRES wakeup

**Table 3:** The distances between the measurement positions and the gateway, the average RSSI [dBm], and the signal quality.

Measurement Positions	Distance (m)	RSSI (dBm)	Signal Quality (%)
P1	4	−34	100
P2	22.2	−55	87
P3	38.5	−72	56
P4	53.6	−80	43
P5	71	−92	13

**Table 4:** Result of test with 5 different wood: Resistance BLE and Moisture BLE are the values transmitted from the device and Moisture meter value is the value read by the MS-7000.

Wood	Resistance (BLE) MΩ	Moisture (BLE) %	Moisture Meter Value %
Pine, Shortleaf	11,730	8.43	8.6
Larch, Western	3980	9.71	9.4
Pine, Red	1570	11.47	11.7
Pine, Ponderosa	39,800	7.23	7.1
Redwood	22,450	7.91	7.7

## Data Availability

Not applicable.
